# Interactive effects of prenatal adversity and COVID-19 hardship on youth psychological distress: a longitudinal study

**DOI:** 10.3389/frcha.2025.1581135

**Published:** 2025-07-17

**Authors:** Yifan Wang, Xinni Wang, Chloé Voyer, Alain Brunet, François Freddy Ateba, Ashley Wazana, David P. Laplante

**Affiliations:** ^1^ School of Psychoeducation, Université de Montréal, Montreal, QC, Canada; ^2^ Centre for Child Development and Mental Health, Lady Davis Institute, Jewish General Hospital, Montreal, QC, Canada; ^3^ State Key Laboratory of Cognitive Neuroscience and Learning, Beijing Normal University, Beijing, China; ^4^ Department of Psychiatry, McGill University, Montreal, QC, Canada; ^5^ National PTSD Research Center, Thompson Institute, University of the Sunshine Coast, Birtinya, QLD, Australia

**Keywords:** COVID-19, mental health, resilience, longitudinal study, early life adversity

## Abstract

**Introduction:**

The COVID-19 pandemic resulted in severe loss of life and increased anxiety as well as fear worldwide. This study explored whether pre-pandemic exposure to varying levels of perinatal maternal adversity coupled with pandemic-related experiences are related to youth distress levels.

**Methods:**

Data from 119 youth (aged 9–17) and their mothers were analyzed to assess the interactive effects of perinatal maternal adversity and pandemic-related objective hardship on youth psychological distress.

**Results:**

Youth-reported hardship models consistently explained more variance in their psychological distress. Youth-reported hardship, specifically daily life changes, predicted psychological distress, including PTSD symptoms and peritraumatic experiences during the pandemic.

**Discussion:**

Youths exposed to high perinatal maternal socio-environmental adversity demonstrated resilience when faced with pandemic disruptions, suggesting that alignment between early adversity and later stress can mitigate distress during crises.

## 1 Introduction

The COVID-19 pandemic led to severe loss of life, major disruption to essential health services, and widespread anxiety and panic across the world. As of January 2025, the World Health Organization reported over seven million COVID-19-related deaths (1). Public health measures, such as social distancing and school closures, were implemented worldwide during the pandemic to contain the spread of the virus. These measures, deemed necessary from a public health perspective, resulted in significant disruptions to the daily lives of youth (2). In addition, parental distress generated by the threat of infection and loss of family income during the pandemic also negatively impacted youth outcomes through alterations of family dynamics (3).

Post-traumatic stress disorder (PTSD) emerged as a major concern worldwide following the outbreak of the COVID-19 pandemic (4). Considerable and similar rates of PTSD were reported in individuals who were directly and indirectly exposed to the virus (5). Meta-analysis studies found post-pandemic PTSD prevalences of 22% across all populations (4) and 14% in children and adolescents (6). Peritraumatic distress, the emotional and physiological distress experienced during or shortly after a potentially traumatic event, is associated with the development of PTSD. Younger people were more affected by peritraumatic distress and more likely to report feelings of helplessness than older people during the pandemic (7). Likewise, peritraumatic dissociation, involving altered experiences of time, space, and oneself, is a robust predictor of PTSD.

While the pandemic's impacts on youth mental health are well-documented (8, 9), the role of pre-existing vulnerabilities in shaping these outcomes remains less understood. Some youth experienced psychological distress in response to the pandemic, but others remained resilient. Previous studies have shown that exposure to prenatal depression and/or anxiety (10, 11) and disaster-related prenatal maternal stress (12) can result in increased behavioral problems that persist throughout life. For instance, prenatal maternal affective symptoms predicted children's general psychopathology and specific internalizing problems in children aged between 4 and 8 years (13). In the context of the pandemic, longitudinal studies have found that youth exposure to low levels of perinatal maternal adversity experienced higher levels of general psychopathology when their pandemic-related daily life changes were also high (14). Similarly, individuals who experienced adverse childhood experiences reported greater psychological distress during the pandemic compared to others (15).

A limited number of studies have investigated the extent to which early life adversities relate to youth pandemic-related outcomes, an unprecedented context for studying the interactive effects between perinatal developmental susceptibility and youth distress. Answering this important question requires knowledge of pre-pandemic factors, assessment of COVID-related experiences, and COVID-specific psychological distress assessments. Longitudinal data accounting for a pre-pandemic baseline measure of mental health is lacking (16) as most studies have used cross-sectional designs to assess the impact of the pandemic on children and adolescents. The conclusions drawn from cross-sectional studies are limited as they provide a brief snapshot of a child’s or adolescent’s current mental health status, but cannot answer questions about pathways leading to their current mental health functioning.

The current study explores how perinatal maternal adversity and maternally and/or youth-reported objective hardship during the COVID-19 pandemic could predict psychological distress in 9 to 17-year-olds. Specifically, we ask: (1) Does perinatal maternal adversity and objective hardship predict (main effect and interaction) psychological distress, after controlling for pre-pandemic psychopathology levels, child sex, assessment age, and assessment site? (2) Do maternally and youth-reported COVID-19 pandemic-specific objective hardship have similar or different influences on youth psychological distress? Given prior evidence that perinatal adversity may increase vulnerability to later psychopathology, we hypothesize that perinatal maternal adversity and maternally and/or youth-reported COVID-19 pandemic-specific objective hardship contributed separately or jointly to youth psychological distress during the COVID-19 pandemic. For the purpose of the present study, youth refers to children and adolescents aged between 9 and 17 years.

## 2 Materials and methods

### 2.1 Participants

Participating mother–child dyads were recruited from the Maternal Adversity, Vulnerability, and Neurodevelopment (MAVAN) longitudinal study (17). The goal of the MAVAN study was to examine the influences of the prenatal environment, the psychological wellbeing of the mother, and factors related to the development of the child across childhood and adolescence. Between 2004 and 2014, 576 mother–child dyads were recruited, with assessments during pregnancy; at 6, 12, 18, 36, 48, 60, and 72 months; and at 13 years. Approximately 350 dyads remained active. All active dyads were invited to participate in the study by telephone or email. Among the active dyads, 150 dyads agreed to participate, while 144 dyads completed pandemic-specific online questionnaires between February and July 2021. However, as maternal perinatal adversity data were missing for some mothers (a main predictor variable that is not imputed), the final sample was reduced to 119 children and 144 mothers. The youth (51.26% boys) were recruited from either Hamilton, ON (*n* = 61) or Montreal, QC (*n* = 58), Canada, and were aged 9 to 17 years (*M* = 13.71 years, *SD* = 2.46 years) (Table 1).

**Table 1:** Demographic characteristics of the participants.

Variables	*N*	*M*	SD
Gender			
Male	61		
Female	58		
Site			
Montreal	58		
Hamilton	61		
Youth age	119	13.71	2.46
Youth ethnicity	91		
Indian (%)	1 (1.0)		
African (%)	4 (4.1)		
Caucasian (%)	79 (81.4)		
Latino (%)	1 (1.0)		
Other/mixed (%)	7 (7.2)		
Mother’s age at birth	141	30.74	4.8
Mother’s education level	142		
Primary (%)	5 (0.4)		
Secondary (%)	15 (10.6)		
College/CEGEP (%)	39 (27.5)		
University or higher (%)	83 (58.5)		
Household income	129		
Less than 20,000	9 (0.01)		
20,000–40,000	16 (12.4)		
40,000–60,000	15 (11.7)		
60,000–80,000	19 (14.7)		
80,000–100,000	27 (20.9)		
Child self-rated objective hardship threat	119	5.59	2.50
Child self-rated objective hardship: daily life change	119	10.36	5.12
Child self-rated IES-6 total score	119	6.31	5.89
Child self-rated PDI total score	119	7.72	7.36
Child self-rated PDEQ total score	119	6.53	7.41
Child self-rated COVID-19 psychological distress	119	0.03	1.02
Child pre-pandemic psychopathology	119	−0.05	0.94
A Factor	115	−0.17	0.56
M Factor	119	−0.18	0.90
Mother self-rated objective hardship: daily life change	119	15.55	5.06
Mother self-rated objective hardship: personal threat	119	5.98	2.82

### 2.2 Measures

#### 2.2.1 Psychological distress

Youth self-reported their psychological distress using the 6-item Impact of Events Scale-6 (IES-6) (18), 13-item Peritraumatic Distress Inventory (PDI) (19), and the 10-item Peritraumatic Dissociative Experiences Questionnaire (PDEQ) (20, 21). The youth were asked to complete the PDI and PDEQ based on their worst personal pandemic experience, as no single traumatic event could define the pandemic. The child versions of the PDI and PDEQ have both demonstrated very good internal consistency (*α* = 0.80 and *α* = 0.77, respectively) (22). The IES-6 has good reliability (*α* = 0.76) (23).

Composite youth psychological distress scores were computed using principal component analysis (PCA) with the IES-6, PDI, and PDEQ scores. The PCA identified a single factor that explained 72.50% of the total variance in the youths' psychological distress scores [psychological distress = [0.36 × IES-6] + [0.40 × PDI] + [0.42 × PDEQ]]. The psychological distress scores were standardized to a mean of 0 and a standard deviation of 1. Positive scores were indicative of psychological distress above the average, while negative scores indicated levels below the average. This approach is consistent with previous disaster studies (24, 25).

#### 2.2.2 Objective Hardship

COVID-related experiences were assessed using a modified version of the Objective Hardship Questionnaire, originally developed for research with pregnant women (26). The current questionnaire on objective hardship tapped into two dimensions: daily life change and personal threat. The goal of the measurement was to obtain a detailed description of stressful events experienced by youth and their mothers without asking whether they had perceived the event to be positive or negative.

The daily life change dimension measured the degree to which the youths' or mothers' daily lives were disrupted by the pandemic. Daily life changes can impact the lives of youth and mothers uniquely. Change levels were computed as the magnitude to represent the overall disruptions rather than the perceived negative or positive (direction of change) effects these changes may have had on their daily lives. For example, the *Much less than normal* or *Much more than normal* responses were considered higher-level changes and scored as 2, and the *Somewhat less than normal* or *Somewhat more than normal* responses were considered lower-level changes and scored as 1. The reason for this approach was that certain changes, such as spending more time with family members, may have been perceived by some individuals as positive, but negative by others.

The personal threat dimension measured the degree of pandemic-related risk to which the youth, mothers, family members, and/or close friends were exposed during the pandemic. For instance, the following question asked about the availability of protective measures at work: “If you worked during the COVID-19 outbreak, were protective measures (e.g., face masks/shields, gloves, gowns) available?”. Another question asked whether the youth or the mother was infected by the COVID-19 virus: “Were you diagnosed with COVID-19?”.

Individual items for each dimension were weighted and summed to form individual continuous scores. There was no attempt to make the subscales numerically equivalent. Theoretically, the daily life change scores ranged from 0 to 28 and those of personal threat from 0 to 29. Separate scores were obtained for the youths and their mothers.

#### 2.2.3 Perinatal maternal adversity

The prenatal maternal affective symptoms factor (M Factor) measured maternal depression and anxiety symptoms during pregnancy. The M Factor was created using 24 items from the 20-item Center for Epidemiologic Studies Depression Scale (Cronbach's *α* = 0.88) (27) and the 4-item Pregnancy-Specific Anxiety scale (*α* = 0.74) in the second trimester (28) and (*α* = 0.77) in the third trimester. Confirmatory factor analyses (CFA) were sequentially used to identify the best fitting and parsimonious model(s). The best model specified one general and three specific latent factors: general affective symptoms (M Factor), specific anxiety/depression (e.g., feelings of anxiousness, worry, depression, and/or loneliness), specific somatic symptoms (e.g., having poor appetite or feeling sick), and pregnancy-specific fears (e.g., feeling concerned about the pregnancy) (13). These measures were harmonized and standardized across all cohorts in the current study.

The perinatal maternal socio-environmental adversity factor (A Factor) refers to a measure of maternal adversity assessed from pregnancy to 6 months postpartum. The construction of the A Factor was based on the cumulative environmental risk score, which has been described elsewhere (29, 30). To compute the A Factor, risk items were first loaded onto four conceptually distinct but related risk domains, and then the four risk domains were loaded onto an overall A Factor producing a higher-order (hierarchical) general A Factor and four specific lower-order factors: (1) stressful life events (i.e., death in family, accident, and/or illness); (2) contextual risk (i.e., poor housing conditions and/or financial problems); (3) parental risk (i.e., alcohol and substance abuse and/or criminal involvement); and (4) interpersonal risk (i.e., family conflict and/or domestic violence). These measures were harmonized across all cohorts in the current study.

#### 2.2.4 Covariates

To control for pre-COVID-19 pandemic levels of youth psychopathology, pre-COVID-19 pandemic child general psychopathology was included as a covariate (31). The latent pre-pandemic child general psychopathology measure included symptom scores obtained from the mother-rated Child Behavior Checklist (Cronbach's *α* = 0.71–0.89) (32), mother-rated Strengths and Difficulties Questionnaire  *α* = 0.71–0.92) (33), mother- and father-rated Conners' Parent Rating Scale (*α* = 0.75–0.94) (34), mother-reported Preschool Age Psychiatrist Assessments (*α* = 0.56–0.89) (35), and Berkeley Puppet Interview (*α* = 0.90–0.99) (36) during childhood. A more detailed description of the pre-pandemic child general psychopathology measure can be found in the work of Sallis and colleagues (31). The youths’ sex and assessment age and assessment site were added to the models as covariates. The assessment site (Montreal or Hamilton) was included as a covariate because the Montreal cohort was primarily comprised of a community-based sample of pregnant women, whereas the Hamilton cohort was a mixture of women with clinically significant depression and/or anxiety during pregnancy and controls. Furthermore, jurisdictional differences in COVID-19 pandemic-related mitigation measures could have affected families' exposure to and perception of their objective hardship.

### 2.3 Procedures

Following a description of the study goal, a Research Electronic Data Capture (REDCap) survey link was sent to mothers. Mothers provided consent for themselves and their children. Youth provided assent. Mothers completed the questionnaires regarding themselves and their children while the children completed age-appropriate questionnaires about themselves. Mothers and youth were compensated for their participation.

### 2.4 Data analysis

#### 2.4.1 Main analysis

Descriptive analysis of the sample was conducted using SPSS 26. To predict COVID-19 pandemic-related psychological distress, the R package LEGIT (Latent Environmental and Genetic InTeraction) (11) was used to model the two-way interactions between perinatal maternal adversity and objective hardship on youth psychological distress. LEGIT was designed specifically to test two- or three-way interactions containing multiple genes and/or environments by using weighted scores for each. Using an alternating optimization method in which weights for genes and environment are estimated in turn by holding all other parameters constant, latent G and E terms are derived based on the best fitting model [the one with the lowest Akaike information criterion (AIC)] after multiple iterations. For each element used to create a latent G or latent E, the weight of the relative contribution of that element to the latent score is also given (e.g., the weight and significance of each included genetic term used to create the latent G). Interaction effects are estimated by holding the previously determined weighted scores constant, creating a G × E term (11). This method significantly reduces the number of interaction parameters compared to traditional methods, which would require a term for each genetic component interacting with each environmental term and thus having much less power. In the present study, LEGIT estimated optimal weights for objective hardship (combining the daily life change and personal threat dimensions). Interaction terms were assessed using *F*-tests, and covariates (the youths’ sex, age, and pre-pandemic general psychopathology, and assessment site) were included as fixed effects. Specifically, a set of two-way models with a perinatal maternal adversity × objective hardship interaction was fitted for psychological distress, where maternal adversity consisted of either the A or M Factor, and objective hardship was a latent factor consisting of the daily life change and personal threat scores. Sex, age, assessment site (Hamilton or Montreal), and pre-COVID child general psychopathology were included as covariates. The A Factor was included as a covariate in models where the M Factor was the predictor. The M Factor was included as a covariate in models where the A Factor was the predictor. Two models were defined: (1) mother-reported objective hardship predicting youth psychological distress, and (2) youth-reported objective hardship predicting youth psychological distress. Exploratory follow-up analyses were conducted on the individual components of the psychological distress composite score (e.g., IES-6, PDI, and PDEQ) only if either the main effect of perinatal maternal adversity, objective hardship, or the perinatal maternal adversity × objective hardship interaction was statistically significant. No correction for multiple hypothesis testing was used, as this work is considered exploratory and for the purpose of generating new hypotheses.

#### 2.4.2 Missing data

Missing data were handled using multiple imputation by chained equations (mice R package; version 3.16.0) for all variables, except for the A and M Factors, if at least one COVID-19 pandemic-related data point was available. The random forest algorithm was used for its robustness when handling diverse data types and its capability of handling complex relationships across 40 imputed datasets to preserve the distributional integrity of the original data and mitigate potential biases in subsequent analyses. The reliability of the imputation was verified through diagnostic plots.

## 3 Results

For all subsequent analyses, LEGIT models were fit to test how psychological distress was associated with perinatal maternal adversity (M Factor or A Factor), maternal (or youth)-reported objective hardship (onto which daily life change and personal threat were loaded), and the interaction of perinatal maternal adversity (M Factor or A Factor) and maternally or youth-reported objective hardship. The following covariates were included in all analyses: the youths’ sex and assessment age, assessment site, pre-pandemic child general psychopathology, and A Factor (when M Factor was the predictor) or M Factor (when A Factor was the predictor).

The primary analyses are stratified by the reporter: one set uses mother-reported objective hardship, while the other set uses youth-reported objective hardship. When significant main effects and/or interactions were observed, exploratory follow-up analyses decomposing the psychological distress composite into its subscales (IES-6, PDI, and PDEQ) were conducted to identify which specific symptoms drive the effects. When significant objective hardship main effects or interactions involving objective hardship were observed, the results indicate which measure (daily life change, personal threat, or both) best defined the objective hardship latent factor. Only significant results are presented. The full models can be found in the Supplementary Material.

### 3.1 Descriptive statistics

Demographic data of the families are presented in Table 1, in which we provide the means and standard deviations of all the variables included in the analysis. Using the PDI score cut-off of 14, 16.8% of the youth met the criteria for potential PTSD (37). Using the IES-6 score cut-offs of 7 (possible) and 9 (probable), 32.8% of youth reported possible PTSD and 26.9% probable PTSD (18). However, none of the youth who met the clinical criteria for potential PTSD believed that they were in danger of losing their life (a necessary Diagnostic and Statistical Manual of Mental Disorders, Fifth Edition, criterion for a PTSD diagnosis).

### 3.2 Mother-reported Objective Hardship as the moderator

#### 3.2.1 Prenatal affective symptoms (M Factor) as the predictor

Girls (*p* = 0.001), youth from Hamilton (*p* = 0.003), and older youth (*p* = 0.003) experienced higher psychological distress. The model explained 22.0% of the variance in psychological distress.

#### 3.2.2 Perinatal social-environmental adversity (A Factor) as the predictor

**Figure 1:**
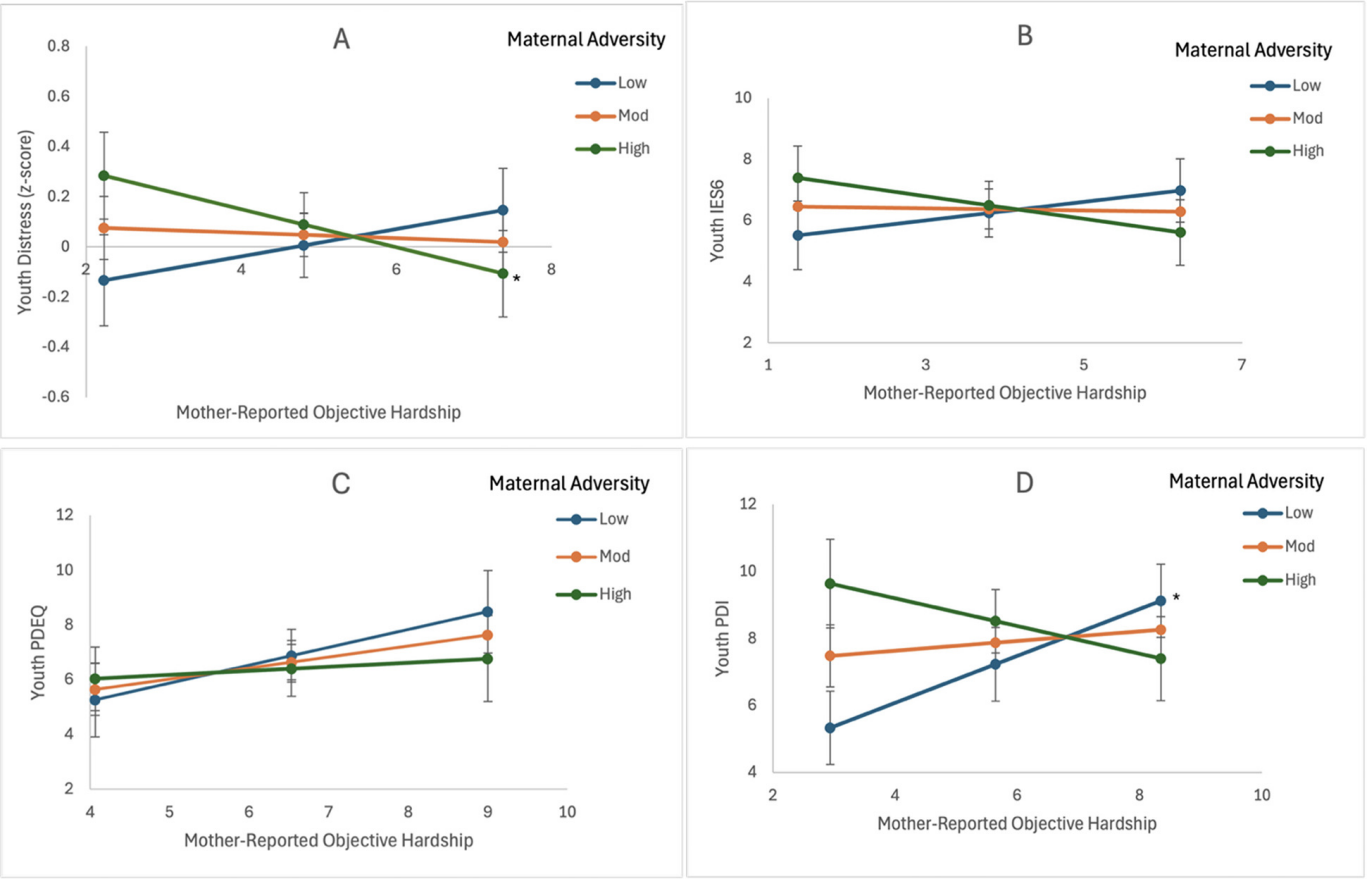
Effect of perinatal social-environmental adversity (A Factor) and mother-reported objective hardship on youth outcomes. Positive slopes indicate increased distress with higher hardship among low-A-Factor youth, while negative slopes suggest attenuation among high-A-Factor youth. **(A)** For youth exposed to high A Factor, psychological distress diminished as maternal objective hardship increased. **(B)** For youth exposed to higher A Factor, PTSD symptoms diminished as maternal objective hardship increased. **(C)** Higher PDEQ is associated with higher maternal objective hardship. **(D)** For youth exposed to low A Factor, peritraumatic distress diminished as maternal objective hardship decreased (*p < 0.05).

The maternal objective hardship latent factor was associated with psychological distress, with personal threat (*p* = 0.014) being the most significant component of objective hardship. A higher score on the A Factor was also associated with higher psychological distress (*p* *=* 0*.*048). The interaction of the A Factor and maternal objective hardship (personal threat) was significant (*p* *=* 0*.*028). The effect of the A Factor on psychological distress diminished as maternal objective hardship (personal threat) increased (Figure 1). Girls (*p* *=* 0.001), youth from Hamilton (*p* *=* 0.003), and older youth (*p* *=* 0.002) also reported higher psychological distress. The model explained 25.0% of the variance in psychological distress.

### 3.3 Exploratory analyses of the effect of perinatal social-environmental adversity (A Factor) and maternal-rated Objective Hardship on youth psychological distress dimensions

#### 3.3.1 PTSD symptoms (IES-6) as the outcome

There was no separate association between maternal objective hardship or the A factor and PTSD symptoms. However, the interaction of the A Factor and the maternal objective hardship latent factor was marginally significant (*p* *=* 0*.*078), with personal threat being the most significant component (*p* = 0.051). Youth exposed to higher A Factor tended to experience lower PTSD symptoms as maternal objective hardship increased. The PTSD symptoms of youth exposed to a low-to-moderate A Factor level did not change as a function of maternal objective hardship (Figure 1). Girls (*p* *=* 0*.*009) and older youth (*p* *=* 0*.*007) also reported more PTSD symptoms. The model explained 16.2% of the variance in PTSD symptoms.

#### 3.3.2 Peritraumatic dissociative experiences (PDEQ) as the outcome

Girls (*p* *<* 0.001), older youth (*p* = 0.016), youth from Hamilton (*p* = 0.004), and youth with higher pre-COVID-19 pandemic general psychopathology (*p* = 0.039) reported more peritraumatic dissociative experiences. The model explained 22.5% of the variance in peritraumatic dissociative experiences.

#### 3.3.3 Peritraumatic distress (PDI) as the outcome

The A Factor, not maternal objective hardship, was separately associated with peritraumatic distress (*p* *=* 0*.*010). Furthermore, the interaction of the A Factor and the maternal objective hardship latent factor was significant (*p* *=* 0*.*009), with daily life change being the most significant component (*p* *=* 0*.*004). For youth exposed to low A Factor levels, their peritraumatic distress was lower than other youth when maternal objective hardship (daily life change) was low (3.79 and lower) (Figure 1). Peritraumatic distress did not change as a function of maternal objective hardship for youth exposed to moderate or high A Factor levels. Youth from Hamilton (*p* *=* 0*.*002) and older youth (*p* *=* 0*.*025) also reported higher peritraumatic distress. The model explained 21.7% of the variance in peritraumatic distress.

### 3.4 Youth-reported Objective Hardship as the moderator

#### 3.4.1 Prenatal affective symptoms (M Factor) as the predictor

**Figure 2:**
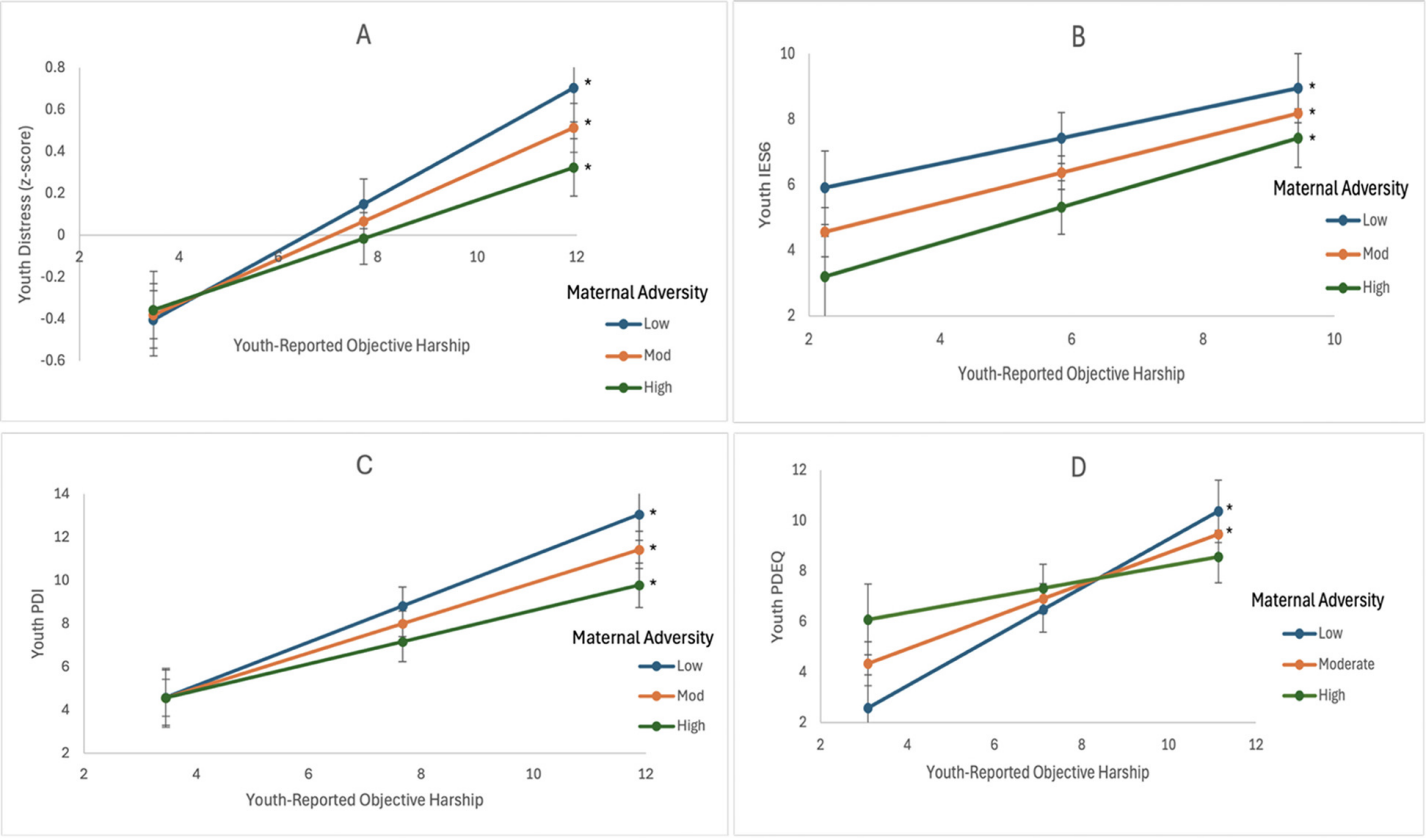
Effect of prenatal affective adversity (M Factor) and youth-reported objective hardship on youth outcomes. Positive slopes indicate increased distress with higher hardship among low-M-Factor youth, while negative slopes suggest attenuation among high-M-Factor youth. **(A)** Higher youth objective hardship is associated with higher youth psychological distress. **(B)** Higher youth objective hardship is associated with more PTSD symptoms. **(C)** Higher youth objective hardship is associated with higher peritraumatic distress. **(D)** For youth exposed to low or moderate M factor levels, peritraumatic dissociative experiences increased as youth objective hardship increased (*p < 0.05).

The youth objective hardship latent factor was associated with psychological distress (*p* *<* 0*.*001), with daily life change being the most significant component of objective hardship. Girls (*p* *=* 0*.*005) and youth from Hamilton (*p* *=* 0*.*036) reported higher psychological distress. The model explained 37.9% of the variance in psychological distress (Figure 2).

#### 3.4.2 Perinatal social-environmental adversity (A Factor) as the predictor

The youth objective hardship latent factor was associated with psychological distress *(p* < 0.001), with daily life change being the most significant component of objective hardship. Higher youth objective hardship was associated with higher psychological distress (*p* < 0.001). Girls (*p* = 0.004), youth from Hamilton (*p* = 0.037), and older youth (*p* = 0.027) reported higher psychological distress. The model explained 37.32% of the variance in youth psychological distress.

### 3.5 Exploratory analyses of the effect of perinatal maternal adversity and youth-rated Objective Hardship on youth psychological distress dimensions

#### 3.5.1 Youth-reported PTSD symptoms (IES-6) as the outcome

##### 3.5.1.1 Prenatal affective symptoms (M Factor) as the predictor

The youth objective hardship latent factor was associated with PTSD symptoms, with daily life change being the most significant component of objective hardship (*p* < 0.001). Higher youth objective hardship was associated with more PTSD symptoms (*p* = 0.001). Girls reported more PTSD symptoms (*p* = 0.038). The model explained 22.3% of the variance in PTSD symptoms (Figure 2).

##### 3.5.1.2 Perinatal social-environmental adversity (A factor) as the predictor

The youth objective hardship latent factor was associated with PTSD symptoms (*p* *<* 0.001), with daily life change being the most significant component of objective hardship. Higher youth objective hardship was associated with more PTSD symptoms (*p* < 0.001). Girls also reported more PTSD symptoms (*p* = 0.026). The model explained 22.5% of the variance in PTSD symptoms.

#### 3.5.2 Youth-reported PDI score as the outcome

##### 3.5.2.1 Prenatal affective symptoms (M Factor) as the predictor

The youth objective hardship latent factor was associated with peritraumatic distress (*p* *<* 0.001), with daily life change being the most significant component of objective hardship. Higher youth objective hardship was associated with higher peritraumatic distress (*p* < 0.001). Youth from Hamilton (*p* = 0.030) also reported higher peritraumatic distress. The model explained 34.7% of the variance in peritraumatic distress (Figure 2).

##### 3.5.2.2 Perinatal social-environmental adversity (A Factor) as the predictor

The youth objective hardship latent factor was associated with peritraumatic distress, with both daily life change (*p* *<* 0.001) and youth personal threat (*p* = 0.075) being significant components of objective hardship. Higher youth objective hardship was associated with higher peritraumatic distress (*p* < 0.001). Youth from Hamilton (*p* = 0.030) reported higher peritraumatic distress. The model explained 34.0% of the variance in peritraumatic distress.

#### 3.5.3 Youth-reported peritraumatic dissociative experiences (PDEQ) as the outcome

##### 3.5.3.1 Prenatal affective symptoms (M Factor) as the predictor

The youth objective hardship latent factor was associated with peritraumatic dissociative experiences (*p* *<* 0.001), with daily life change being the most significant component of objective hardship. Higher youth objective hardship was associated with more peritraumatic dissociative experiences (*p* < 0.001). The interaction of the M Factor and youth objective hardship was significant (*p* = 0.019). Youth exposed to low or moderate M Factor levels reported more peritraumatic dissociative experiences as their objective hardship increased. The peritraumatic dissociative experiences of youth exposed to low or moderate M Factor levels significantly differed from youth exposed to high M Factor levels when their objective hardship was either low (−0.007 and lower) or high (14.38 and higher). The peritraumatic dissociative experiences of youth exposed to high M Factor levels did not change as a function of their objective hardship (Figure 2). Girls (*p* = 0.001) and youth with higher pre-COVID-19 pandemic general psychopathology levels (*p* = 0.023) reported more peritraumatic dissociative experiences. The model explained 32.6% of the variance in peritraumatic dissociative experiences.

##### 3.5.3.2 Perinatal social-environmental adversity (A factor) as the predictor

The youth objective hardship latent factor was associated with peritraumatic dissociative experiences (*p* *<* 0.001), with daily life change being the most significant component of objective hardship. Higher objective hardship was associated with more peritraumatic dissociative experiences (*p* < 0.001). Girls (*p* = 0.002) and older youth (*p* = 0.004) also reported more peritraumatic dissociative experiences. The model explained 32.8% of the variance in peritraumatic dissociative experiences.

## 4 Discussion

The present study examined the interplay between youth exposure to perinatal maternal adversity and COVID-19 pandemic-related youth- and mother-reported objective hardship, encompassing daily life change and personal threat, in predicting youth COVID-19 pandemic-related psychological distress. These analyses were strengthened by a longitudinal design, measurement of adversity in early life, a robust characterization of COVID-19 pandemic adversity, and a detailed assessment of psychopathology. The models tested in the present study explained 22.0%–37.9% of the variance in youth psychological distress, depending on who reported the objective hardship (maternal vs. youth) and the distress subdomain. Notably, youth-reported objective hardship models consistently explained more variance in their own distress.

Across all the models tested, girls consistently presented higher psychological distress compared to boys. Our results corroborate other Canadian studies reporting higher perceived stress, depression symptoms, and anxiety among adolescent girls during the COVID-19 pandemic (38). Youth age was also associated with psychological distress, with older children demonstrating higher levels of distress during the COVID-19 pandemic. Considering that the oldest youth in this study was 17 years old, it is possible that they were experiencing more disruption in their daily lives and were more aware of the severity of the disease than their younger counterparts (39).

### 4.1 Perinatal maternal adversity

In the present study, perinatal maternal adversity was not consistently associated separately or jointly with youth outcomes during the COVID-19 pandemic. For instance, prenatal affective symptoms (M Factor) were not associated with youth psychological distress in any of the models tested, suggesting that they either had no effect or a limited direct effect on youth mental health functioning during the COVID-19 pandemic. In contrast, perinatal maternal socio-environmental adversity (A Factor) was positively associated with youth overall psychological distress and peritraumatic distress in models with mother-reported objective hardship. This finding suggests that socio-environmental risks during the prenatal period may exert a stronger and more direct influence on youth distress during periods of crises compared to prenatal maternal mood and anxiety problems. This is in line with earlier studies linking maternal psychosocial stress during pregnancy and child social-emotional development (40, 41). We propose that the stronger predictive power of socio-environmental adversity can be attributed to the potential chronicity and/or stability of adverse environmental factors. Socio-environmental adversities, such as financial instability, poor housing conditions, or family conflict, tend to persist beyond the prenatal period, creating a cumulative risk environment for the child, which may have been exacerbated during the subsequent periods of prolonged stress that were observed during the pandemic. Whereas affective symptoms during pregnancy, while shown to be predictive of later youth outcomes (13) and even COVID-19 pandemic-related psychopathology (14), tend to fluctuate over time (42), and may have less of an overall impact on youth crisis-specific distress during periods of high global stress. Given that we found that 25% of the variance was explained by maternal socio-environmental adversity, interventions targeting family-level hardships could mitigate up to one-quarter of pandemic-related distress in youth.

### 4.2 Objective Hardship

The outbreak of the COVID-19 pandemic brought drastic changes across many domains of daily life and posed threats to youth health. We found that youth self-reported objective hardship was positively associated with their psychological distress. Specifically, these associations were primarily driven by the daily life change dimension, which documented the degree to which youths' daily lives were disrupted by the COVID-19 pandemic and its associated mitigation measures. Youth are attuned to disruptions in their daily routines as their sense of stability is largely anchored to consistent activities such as going to school and playing with friends. However, the personal threat dimension did not significantly contribute to their psychological distress. This suggests that the COVID-19 pandemic-related life threat events potentially experienced by the youth in this study may be only a small component of their experienced hardships, while the shift in their routines precipitated by the COVID-19 pandemic had a stronger impact on their psychological distress. Policies should therefore focus on stabilizing routine disruptions, which accounted for 34.7% of the variance in peritraumatic distress.

Mothers were also asked to report on their own COVID-19 pandemic experiences, and the effects of mother-reported objective hardship on youth psychological distress were primarily driven by the personal threat dimension, which indicated the degree of COVID-19 pandemic-related risk to which the youths, mothers, or family members were exposed. The observed discrepancies between the associations between maternally and youth-reported objective hardship and youth psychological distress suggest that the COVID-19 pandemic was experienced differently within the family system. From the mothers' perspectives, the threat stemming from the COVID-19 pandemic (e.g., the virus) was the most important dimension in the association between the objective hardship and youth psychological distress. We also speculate that the youth in this study were potentially more aware and responsive to their mothers' COVID-19 pandemic-related threat of illness and/or death than they were to their mothers' concerns related to daily life disruptions. Furthermore, maternal objective hardship was not associated with any youth-reported outcomes in the present study. These findings suggest that the youths’ self-reported experience of the COVID-19 pandemic may be more important in predicting their own pandemic-related outcomes. While a mother’s report may contribute important information about their child, we suggest that integrating the child's self-assessment can provide a more comprehensive representation of their distress level. COVID-19 pandemic research can benefit from obtaining both parental reports and children's self-assessments to more accurately depict children's outcomes, as the experiences of mothers and youth may have differed.

It should be noted that our scoring of COVID-19 pandemic-related objective hardship focused exclusively on the magnitude of daily life changes and personal threat exposure, rather than their perceived positivity or negativity. While alternative scoring systems incorporating event valence exist (43), we chose this approach because the same event could be appraised as either positive or negative depending on individual circumstances. As such, we focused on disruption severity regardless of direction.

### 4.3 Interaction between youth-reported objective hardship and perinatal maternal adversity

We found that youth self-reported objective hardship moderated the relationship between prenatal maternal affective symptoms (M Factor) and youth COVID-19 pandemic-related psychological distress, particularly their peritraumatic dissociative experiences. For youths exposed to higher prenatal maternal affective symptoms, neither their overall psychological distress nor their peritraumatic dissociative experiences varied as a function of their objective hardship. Conversely, youths exposed to low or moderate prenatal maternal affective symptoms were more sensitive to their COVID-19 pandemic-related objective hardship experiences, as their overall psychological distress, and particularly their dissociative symptoms, increased significantly with increasing objective hardship. For these children, disruptions in daily routines appeared to affect their mental health, manifesting as distorted experiences of time, place, and person (21). While the children exposed to high levels of prenatal maternal affective symptoms also experienced changes in their daily lives, they seemed more resilient to shifting routines and reported fewer dissociative symptoms. This finding is similar to that observed in this and other studies when youth psychopathology was measured: exposure to high levels of perinatal maternal adversity protects youth from the effects of pandemic adversity (44–46). This finding is also in line with previous work, suggesting dissociative symptoms were prevalent among adolescents during the COVID-19 pandemic, as prolonged isolation can increase loneliness and lead to a heightened sense of disconnection from surroundings (47). Another possible explanation for their dissociative experiences is the increased time spent online and gaming among children during the national lockdown (44). Positive associations between excessive gaming, problematic internet usage, and dissociative experiences in adolescents have been previously reported (48, 49). While not directly assessed in the present study, it has been shown that immersion in the virtual world can turn into a state of disconnection from reality. As players lose awareness of their surrounding environment, they tend to disconnect from their feelings, thoughts, and behavior (48).

### 4.4 Interaction between mother-reported objective hardship and perinatal maternal adversity

Our study also asked mothers to report on their own experiences, and these experiences were used as a proxy for household objective hardship levels. Mother-reported objective hardship was not directly associated with youth psychological distress. However, for youth exposed to low or moderate maternal socio-environmental adversity (A Factor), their psychological distress increased as mother-reported objective hardship (driven primarily by personal threat) increased. When the dimensions of the youth composite psychological distress outcome were analyzed separately, this interactive effect was observed for youth self-reported peritraumatic distress and to a lesser extent for youth-reported PTSD symptoms. These findings suggest that youth from households with fewer problems (i.e., less marital strain, fewer monetary problems, fewer major disruptive events, and better-educated mothers), at least during the perinatal period, experienced an overall increase in psychological distress. In particular, higher peritraumatic distress was observed when mothers reported experiencing higher COVID-19 pandemic-related experiences that threatened them or their family members' personal safety. This finding suggests that the personal safety threats due to the COVID-19 pandemic differentially impacted youth as a function of the household's perinatal socio-environmental adversity status.

Individual differences in reaction toward COVID-19 pandemic-related stressors align with the developmental-mismatch hypothesis (50), which posits that a mismatch between one’s early and adult environments results in increased risk for mental health challenges. When there is a match between the two, decreased risk is expected. For youth exposed to high levels of prenatal adversity, the stressful pandemic environment was consistent with their early environment, and such continuity may mitigate the adverse effects of later life stress. While routine changes during the pandemic were stressful, it seems that they were not sufficiently stressful to create a mismatch in stress levels; thus, we did not observe increased dissociative symptoms. In contrast, youth exposed to low-to-moderate prenatal adversity may have been habituated to relatively stable and low-stress environments. These youth may have lacked the adaptive flexibility needed to navigate stressful events, making them more susceptible to dissociation (50).

The moderating effects of objective hardship seem closely tied to how mothers and youth experience and interpret hardship. Mothers, as primary caregivers, often prioritize the personal threat dimension, focusing on tangible risks such as health and safety, while youths are more affected by disruptions in their daily routines. Guided by Carr's family systems theory, stressors that impede a mother’s functioning can ripple through the family system, influencing their children's adjustment in a cascading way (51). We hypothesize that when mothers perceive heightened COVID-19 pandemic-related life threats for their family, their distress might have cascading effects on their children, resulting in the children reporting more peritraumatic distress and dissociative symptoms. Maternal reporting of heightened COVID-related threats likely increases their distress, which can manifest in changes to parenting behavior or reduced capacity to provide emotional support. These disruptions in maternal caregiving may amplify children's vulnerability to psychological distress, particularly under conditions of high perinatal socio-environmental adversity.

### 4.5 Limitations and strengths

This study has limitations. The sample was relatively small and primarily represented a Caucasian population, limiting the generalizability of findings to other cultural or socio-economic contexts. The study, however, has several strengths. The first strength lies in the use of a longitudinal design. Much of the research to date on children's mental health functioning during the COVID-19 pandemic has used cross-sectional data. The available comprehensive perinatal measures of maternal mood and socio-environmental factors in this study provided a rare opportunity to examine the interplay between early risk factors and external stressors during a global crisis. The use of LEGIT and latent factors circumvented, to a degree, the power issues associated with smaller samples by reducing the overall number of analyses. Moreover, the composite psychological distress score (our main outcome) also reduced the overall number of analyses required but allowed for follow-up exploratory analyses on its components when appropriate. Finally, we add to the literature by arguing for the inclusion of youth self-reports of their own experiences and functioning when considering pathways from perinatal maternal adversity to youth mental health functioning.

## 5 Conclusion

The COVID-19 pandemic disrupted every aspect of life on a global scale, but not all children were affected equally. As supported by our findings, youth exposed to greater disruption to their daily routine during the pandemic reported greater psychological distress during the COVID-19 pandemic. While the COVID-19 pandemic was perceived as a threat to personal safety by all participating families, its effect was insufficient to affect the psychological distress levels in youth who were born in households with socio-environmental adversities or youth exposed to high prenatal maternal affective symptoms. Importantly, early adversities could paradoxically confer resilience for some youth, particularly when the subsequent stressors mirrored the adverse conditions of their early environment. Our findings can inform targeted prevention and early interventions, which can mitigate the development of later psychopathology.

## Data Availability

The raw data supporting the conclusions of this article will be made available by the corresponding authors on request. Requests to access these data should be directed to david.laplante@ladydavis.ca or ashley.wazana@mcgill.ca.
