# Supplementary material for: Interactive effects of prenatal adversity and COVID-19 hardship on youth psychological distress: a longitudinal study
Source: Front Child Adolesc Psychiatry. 2025 Jul 17;4:1581135. doi: 10.3389/frcha.2025.1581135 (PMC12310598; doi:10.3389/frcha.2025.1581135)
Supplement: Supplementary file 1 [file Datasheet1.docx]

Supplementary Material

**Results**

**Mother-Reported Objective Hardship as Moderator**

***Prenatal Affective Symptoms (M Factor) as Predictor***

We fit a LEGIT model to test how youth Psychological Distress was associated with the M-factor, maternal Objective Hardship (onto which Daily Life Change and Personal Threat were loaded), M-factor by maternal Objective Hardship and covariates. Neither the M Factor, maternal Objective Hardship, nor the M Factor × maternal Objective Hardship interaction was significant. Females (*p* = .001), Hamilton youth (*p* *= .*003), and older youth (*p* *=.*003) experienced higher youth Psychological Distress. The model explained 21.96% of variance of youth Psychological Distress.

***Perinatal Social-Environmental Adversity*** ***(A Factor) as Predictor***

Personal Threat (*p*= .014) contributed to the maternal Objective Hardship. Higher Perinatal Social-Environmental Adversity (A Factor) was associated with higher youth Psychological Distress (*p = .*048) while maternal Objective Hardship was not significant. The A Factor × maternal Objective Hardship interaction was significant (*p = .*028). The effect of A Factor on youth Psychological Distress diminished as maternal Objective Hardship increased (Figure 1). Females (*p =*.001), Hamilton youth (*p =*.003), and older youth (*p =*.002) reported higher Psychological Distress. The model explained 25.02% of variance of youth Psychological Distress.

**Exploratory Analyses of the Effect of Perinatal Social-Environmental Adversity** **(A Factor) and Maternal Objective Hardship on Youth Psychological Distress Dimensions.**

***Youth reported PTSD Symptoms (IES-6) as Outcome***

Personal Threat (*p*= .051) contributed to the maternal Objective Hardship. The A Factor and maternal Objective Hardship were nonsignificant. The A Factor × maternal Objective Hardship interaction was marginally significant (*p = .*078). Females (*p = .*009) and older youth (*p* *= .*007) reported higher PTSD symptoms. The model explained 16.24 % of variance of the youths’ PTSD symptoms. **Slope Analysis**. Youth exposed to higher Perinatal Social-Environmental Adversity (A Factor) tended to experience lower PTSD symptoms as maternal Objective Hardship scores increased. PTSD symptoms of youth exposed to low to moderate levels of Perinatal Social-Environmental Adversity (A Factor) did not change as a function of maternal Objective Hardship (Figure 1).


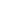

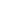


***Youth Peritraumatic Dissociative Experiences (PDEQ)*** ***as Outcome***

Neither Personal Threat nor Daily Life Change significantly contributed to the maternal Objective Hardship. Neither the A Factor, maternal Objective Hardship, nor the A Factor × maternal Objective Hardship interaction was significant. Females (*p <*.001), older youth (*p*= .016), Hamilton youth (*p* = .004), and youth with higher pre-COVID General Psychopathology (*p*=.039) reported higher Peritraumatic Dissociative Experiences. The model explained 22.48% of variance of youth Peritraumatic Dissociative Experiences.

***Youth Peritraumatic Distress Inventory (PDI) as Outcome***

Daily Life Change (*p = .*004) contributed to the maternal Objective Hardship. Higher Perinatal Social-Environmental Adversity (A Factor) was associated with higher youth Peritraumatic Distress (*p = .*010) while maternal Objective Hardship was not significant. The A Factor × maternal Objective Hardship interaction was significant (*p = .*009). Hamilton youth (*p = .*002) and older youth (*p = .*025) reported higher Peritraumatic Distress. The model explained 21.69% of variance of youth Peritraumatic Distress. **Slope Analysis**. Figure 1 shows Peritraumatic Distress differed as a function of Perinatal Social-Environmental Adversity (A Factor) and maternal Objective Hardships only for youth exposed to low Perinatal Social-Environmental Adversity (A Factor). Their peritraumatic distress was lower than youth exposed to high Perinatal Social-Environmental Adversity (A Factor) when maternal Objective Hardship was low (3.79 and lower). Peritraumatic distress did not change as a function of maternal objective hardship for youth exposed to moderate or high Peritraumatic Distress Perinatal Social-Environmental Adversity (A Factor).

**Youth-Reported Objective Hardship as Moderator**

***Prenatal Affective Symptoms (M Factor) as Predictor***

Daily Life Change (*p <*.001) contributed to the youth Objective Hardship. Higher youth Objective Hardship was associated with higher Psychological Distress (*p < .*001) while the M Factor was nonsignificant. The M Factor × youth Objective Hardship interaction was not significant. Females (*p = .*005) and Hamilton youth (*p = .*036) reported higher Psychological Distress. The model explained 37.94% of variance in youth Psychological Distress (Figure 2).

***Perinatal Social-Environmental Adversity*** ***(A Factor) as Predictor***

Daily Life Change (*p <* .001) contributed to the youth Objective Hardship. Higher youth Objective Hardship was associated with higher youth Psychological Distress (*p < .*001) while the A Factor was nonsignificant. The A Factor × youth Objective Hardship interaction was not significant. Females (*p = .*004), Hamilton youth (*p = .*037), and older youth (*p = .*027) reported higher youth Psychological Distress. The model explained 37.32% of variance in youth Psychological Distress.

**Exploratory Analyses of the Effect of Perinatal Maternal Adversity and Youth Objective Hardship on Youth Psychological Distress Dimensions.**

***Youth-Reported PTSD Symptoms (IES6) as Outcome***

**Prenatal Affective Symptoms (M Factor) as Predictor**. Daily Life Change significantly (*p <*.001) contributed to the youth Objective Hardship latent score. Higher youth Objective Hardship was associated with higher PTSD symptoms (*p*= .001) while the M Factor was nonsignificant. The M Factor × youth Objective Hardship was not significant. Females reported more PTSD symptoms (*p*= .038). The model explained 22.27% of variance of youth PTSD symptoms (Figure 2).

**Perinatal Social-Environmental Adversity** **(A Factor) as Predictor**. Daily Life Change (*p <*.001) contributed to the youth Objective Hardship latent score. Higher youth Objective Hardship was associated with higher PTSD symptoms (*p*< .001) while the A Factor was nonsignificant. The A Factor × youth Objective Hardship was not significant. Females reported higher PTSD symptoms (*p*= .026). The model explained 22.46% of variance of youth PTSD symptoms.

***Youth-Reported Peritraumatic Distress Inventory (PDI) as Outcome***

**Prenatal Affective Symptoms (M Factor) as Predictor**. Daily Life Change (*p <*.001) contributed to the youth Objective Hardship. Higher youth Objective Hardship was associated with higher Peritraumatic Distress (*p*< .001) while the M Factor was nonsignificant. The M Factor × youth Objective Hardship was not significant. Hamilton youth (*p*= .030) reported higher Peritraumatic Distress. The model explained 34.72% of variance of youth Peritraumatic Distress (Figure 2).

**Perinatal Social-Environmental Adversity** **(A Factor) as Predictor**. Daily Life Change (*p <*.001) and Youth Personal Threat (*p* = .075) contributed to the youth Objective Hardship. Higher youth Objective Hardship was associated with higher Peritraumatic Distress (*p*< .001) while the A Factor was nonsignificant. The A Factor × youth Objective Hardship was nonsignificant. Hamilton youth (*p*= .030) reported higher Peritraumatic Distress. The model explained 34.01% of variance of youth Peritraumatic Distress.

***Youth-Reported Peritraumatic Dissociative Experiences (PDEQ) as Outcome***

**Prenatal Affective Symptoms (M Factor) as Predictor**. Only Daily Life Change (*p < .*001) contributed to youth Objective Hardship. Only the main effect of youth Objective Hardship was significant (*p*< .001): higher youth Objective Hardship was associated with higher Peritraumatic Dissociative Experiences. The M Factor × youth Objective Hardship was significant (*p*= .019). Females (*p*= .001) and youth with higher pre-COVID General Psychopathology levels (*p*= .023) reported higher Peritraumatic Dissociative Experiences. The model explained 32.58% of variance of Peritraumatic Dissociative Experiences.

**Slope Analysis**. Youth exposed to low or moderate levels of Prenatal Affective Adversity (M Factor) reported higher Peritraumatic Dissociative Experiences as their Objective Hardship increased. The Peritraumatic Dissociative Experiences of youth exposed to low or moderate levels of Prenatal Affective Adversity (M Factor) significantly differed from youth exposed to high levels of Prenatal Affective Adversity (M Factor) when their Objective Hardship was either low (−0.007 and lower) or high (14.38 and higher). The Peritraumatic Dissociative Experiences of youth exposed to high levels of Prenatal Affective Adversity (M Factor) did not change as a function of their Objective Hardship (Figure 2).

**Perinatal Social-Environmental Adversity** **(A Factor) as Predictor**. Daily Life Change (*p <*.001) contributed to the youth Objective Hardship latent score. Higher Objective Hardship was associated with more Peritraumatic Dissociative Experiences (*p*< .001): The A Factor × youth Objective Hardship was not significant. The A Factor was nonsignificant. Females (*p*= .002) and older youth (*p*= .004) reported more Peritraumatic Dissociative Experiences. The model explained 32.85% of variance of Peritraumatic Dissociative Experiences.
